# Dexamethasone-Induced Adipose Tissue Redistribution and Metabolic Changes: Is Gene Expression the Main Factor? An Animal Model of Chronic Hypercortisolism

**DOI:** 10.3390/biomedicines10092328

**Published:** 2022-09-19

**Authors:** Flaviane de Fatima Silva, Ayumi Cristina Medeiros Komino, Sandra Andreotti, Gabriela Boltes Reis, Rennan Oliveira Caminhotto, Richardt Gama Landgraf, Gabriel Orefice de Souza, Rogerio Antonio Laurato Sertié, Sheila Collins, Jose Donato, Fabio Bessa Lima

**Affiliations:** 1 Department of Physiology and Biophysics, Institute of Biomedical Sciences, University of Sao Paulo, Sao Paulo 05508-000, Brazil; 2 Department of Pharmaceutical Sciences, Federal University of Sao Paulo, Diadema 09913-030, Brazil; 3 Department of Nutrition Sciences, University of Alabama at Birmingham, Birmingham, AL 35294, USA; 4 Division of Cardiovascular Medicine, Department of Medicine, Vanderbilt University Medical Center, Nashville, TN 37232, USA; 5 Department of Molecular Physiology and Biophysics, Vanderbilt University Medical Center, Nashville, TN 37232, USA

**Keywords:** Cushing’s syndrome, adipose plasticity, visceral obesity, glucocorticoids, dexamethasone, gene expression, lipolysis, lipogenesis, BAT dysfunction, BAT whitening

## Abstract

Chronic hypercortisolism has been associated with the development of several metabolic alterations, mostly caused by the effects of chronic glucocorticoid (GC) exposure over gene expression. The metabolic changes can be partially explained by the GC actions on different adipose tissues (ATs), leading to central obesity. In this regard, we aimed to characterize an experimental model of iatrogenic hypercortisolism in rats with significant AT redistribution. Male Wistar rats were distributed into control (CT) and GC-treated, which received dexamethasone sodium phosphate (0.5 mg/kg/day) by an osmotic minipump, for 4 weeks. GC-treated rats reproduced several characteristics observed in human hypercortisolism/Cushing’s syndrome, such as HPA axis inhibition, glucose intolerance, insulin resistance, dyslipidemia, hepatic lipid accumulation, and AT redistribution. There was an increase in the mesenteric (meWAT), perirenal (prWAT), and interscapular brown (BAT) ATs mass, but a reduction of the retroperitoneal (rpWAT) mass compared to CT rats. Overexpressed lipolytic and lipogenic gene profiles were observed in white adipose tissue (WAT) of GC rats as BAT dysfunction and whitening. The AT remodeling in response to GC excess showed more importance than the increase of AT mass per se, and it cannot be explained just by GC regulation of gene transcription.

## 1. Introduction

Glucocorticoids (GCs) are steroid hormones synthesized and released by cells of the fasciculate zone of the adrenal gland, stimulated by the hypothalamic-hypophyseal-adrenal (HPA) axis in response to several physiologic, environmental, psychological, and stressing stimuli [1]. Cortisol is the main GC in humans and is secreted in a rhythmical circadian pattern of oscillation with more intense peaks of release in the early morning hours [2]. In rodents, the main GC is corticosterone, but with peaks of release at night, due to the nocturnal behavior [3]. This rhythmical synthesis, release, and action are of utmost importance and affect many physiological processes, including regulatory actions on metabolism, growth, development, and inflammatory response [2,4]. The loss of the circadian rhythm and the inhibition of the HPA axis in response to GC excess are the main factors that define Cushing’s syndrome, iatrogenic being the most common [5,6].

Synthetic GC such as dexamethasone is used worldwide as a treatment for several diseases due to its anti-inflammatory and immunosuppressive effects [7,8], with more relevance in the last two years due to massive use as part of COVID-19 treatment, mainly in severe cases, which the patients need hospitalization for long periods [9,10,11]. Compared to other synthetic GCs, dexamethasone has the advantage of low affinity for mineralocorticoid receptors (MR) [12], which could decrease the chances of side effects, since most are a result of the GC-MR binding [13,14]. However, dexamethasone has a higher affinity to the GC receptor (GR) and does not need 11-βHSD1 for its activation, making this GC more potent and increasing the risk of side effects by the GC-GR interaction [12,15].

The classic effects of GC are genomic through GR. The dimeric complex GC-GR is translocated to the nucleus, where the complex can bind the GC responsive elements (GREs) in the regulatory regions of target genes, or act directly by inhibiting or activating other transcription factors, in a GREs-independent pathway [2,16,17]. These bindings can induce or repress gene expression and, consequently, the synthesis of proteins/enzymes that regulate several physiological processes such as glucose homeostasis and lipid metabolism [2,8]. However, these genomic effects require hours or days to happen [18], and in the case of chronic GC exposure, mainly in excess, can result in side effects in many tissues and organs, as observed in Cushing syndrome. In that regard, if there were a way to dissociate these gene expression side effects in other tissues from the anti-inflammatory properties, the risk of complications due to long-term GC exposure would decrease [19].

One of the major characteristics of Cushing’s syndrome is the specific fat accumulation in the central region of the body, such as the abdomen, chest, head, and neck [20,21]. This centripetal fat distribution seems to be due to the hyperplasia and hypertrophy of visceral adipocytes and the differentiation of preadipocytes [22,23,24]. Previous results of our research group did not observe central fat distribution [25]. Other studies using GC excess focused on one or two specific AT territories, or with more emphasis on muscle or liver effects [26,27,28,29]. Considering that central obesity contributes to the development of several metabolic complications [30], and each AT deposit is composed of a distinct subpopulation of adipocytes [31,32] and has a different vascularization and inter-organ drainage [33,34], it becomes important to investigate how GC excess affects the AT in different anatomic locations.

In this work, we present a model of chronic iatrogenic hypercortisolism in young adult rats [35], which can be useful to assess and understand how the GCs excess impacts AT redistribution and plasticity.

## 2. Materials and Methods

### 2.1. Ethics Approval and Animals

All protocols described were approved by the Committee of Ethics in the Use of Animals of the Institute of Biomedical Sciences, University of Sao Paulo (CEUA-ICB/USP #89/2016; #26/2017; 9535190219). For the experiments, SPF-certified 8 weeks old male Wistar Hannover rats obtained from the Institute of Biomedical Sciences Animal Facility were used, since sex could be an important variable in adipose tissue analyzes [36]. Each rat was housed in an individual open polycarbonate cage (model 1291H, 425 × 266 × 185 mm and 800 cm^2^ of floor area, Tecniplast^®^, Buguggiate, VA, Itália Italy) in our laboratory private room at Animal Care of the Department of Physiology and Biophysics for 4 weeks of acclimatization to the new space, light cycle, and human–rat bond before the treatment protocol starts. The room was equipped with a controlled air system (20 air renewal/h and 45–55% humidity), temperature (22 ± 2 °C), and light (12 h light/12 h dark, with lights on at 22h00–reverse cycle, with appropriated infrared light). The rats received standard rodents chow Nuvilab^®^ CR-1 (Nuvital, Colombo, PR, Brazil) and filtered water ad libitum, and these parameters were monitored and replaced twice a week, as well as the cage’s bedding (GOOD LIFE PINUS RG, Granja R.G, Suzano, SP, Brazil) and the enrichment-autoclaved handcrafted cardboard rolls. The cages were always paired in the rack, ensuring the rats could have visual/olfactory contact with the rat(s) in the cage(s) beside.

Only the same two researchers handled the rats during the acclimatization and treatment period for daily monitoring, cleaning, anesthesia protocols, pre and postoperative care, surgery, oGTT, and euthanasia. No external people were allowed in the room to avoid possible external stress, except the Animal Care veterinarian when necessary. The treatment protocol started when rats were young adults (12 weeks old). For treatment, 36 rats were randomly divided into two groups: control (CT, n = 16) and treated with glucocorticoid (GC; n = 20) using the Random Sequence feature (https://www.random.org/sequences/, accessed on 10 July 2017).

### 2.2. Chronic GC Treatment

To ensure continuous and invariable GC administration, an osmotic pump (model 2004, ALZET^®^, Cupertino, CA, USA) was surgically implanted in the dorsal subcutaneous region. The surgeries were realized in an appropriate experimental room at Animal Care. Each rat was weighed and anesthetized with ketamine hydrochloride (100 mg/kg; i.p., Dopalen, Ceva, Sao Paulo, Brazil) and xylazine hydrochloride (5 mg/kg; i.p., Anasedan, Ceva, Sao Paulo, Brazil) and monitored until complete sedation and anesthesia. At this point, we performed the first nasal–anal measure (Day 0). For eyes hydration during the surgery time (~10 min), we used sterile NaCl 0.9% solution. The rats were submitted to trichotomy of the under scapulae region, local asepsis with 10% polyvidone iodine, and placed in the sterile surgical field. The 1.0 cm horizontal incision was performed with a scalpel, a subcutaneous space was opened with a Blunt scissor, and the minipump containing the GC dexamethasone sodium phosphate (sc-204715, Santa Cruz Biotechnology, Dallas, TX, USA) was allocated according to the manufacturer’s instructions. The incision was closed with a continuous suture (4-0, Seda-Silk, Ethicon* by Johnson & Johnson, Sao Jose dos Campos, SP, Brazil), the local was cleaned with 10% polyvidone iodine, and a topic antibiotic ointment was applied (250 U Bacitracin + 3.5 mg Neomycin, Nebacetin^®^, Takeda Pharma, Jaguariuna, SP, Brazil). The rats remained on a tissue-covered 37 °C heated surface (EFF421, INSIGHT^®^, Ribeirao Preto, SP, Brazil), being monitored for vital parameters and ocular hydration until complete recovery from anesthesia. The wound recovery and the rats’ behavior were monitored daily. The antibiotic ointment was applied once a day in the first week, and the suture fell off about 10 days post-surgery. No oral analgesics were administrated as postoperative. CT rats were submitted to the sham surgery. The GC was diluted in sterile 0.9% NaCl solution (6.25 mg in 200 µL). Considering 375 g as the mean initial body weight and the daily amount released by the pump (6 µL), the corresponding daily dose was 0.5 mg/kg per day of GC for 4 weeks. 

The body weight was evaluated on day 0 (mini-pump implantation) and day 28 (euthanasia), and also weekly. The food intake was measured weekly through the difference between the chow offered and the leftover (in grams), expressed as daily food intake per week and as the average of the 28-days daily food intake, both relatives to 100 g of body weight (g/100 g b.w.)

### 2.3. Oral Glucose Tolerance Test (oGTT)

In the last week of treatment and after 8 h of food deprivation, the rats of both groups were submitted to the oral glucose tolerance test (in the nocturnal phase of the light cycle at ZT [zeitgebber time] 22). After topic anesthesia (5% xylocaine gel), blood samples were collected from a 1.0 mm tail tip cut at time 0 (basal glucose and insulin assessment), followed by oral (gavage) administration of glucose 75 mg/100 g of body weight. New blood collections for glucose and insulin measurements were performed at times 5, 10, 15, 30, 45, 60, and 90 min after glucose administration. The blood glucose was assessed in a glucometer (One Touch Ultra^®^, Johnson & Johnson, Sao Jose dos Campos, SP, Brazil). For insulin analysis, the blood collected in heparinized capillary tubes was diluted (1:1; NaCl 0.9% plus heparin 10 IU/mL), centrifuged, and the plasma fraction was utilized for insulin ELISA measurement (#A05105, Bertin Bioreagent, Montigny-le-Bretonneux, France). For the statistical analysis, the areas under curves (AUCs) for blood glucose and insulin were calculated.

### 2.4. Euthanasia

The endpoint was the 28th day of treatment for all animals. Due to reasons beyond our control, the euthanasia could not be performed on the 28th day in two occasions, and six rats (3 from the CT group and 3 from the GC group) were not considered in this study. The final number of rats/samples was 13 for the CT group and 17 for the GC group. 

The rats from both groups were submitted to 12 h of food deprivation and euthanized during the first hour after lights off (1000–1100 h, 12 < ZTs < 13) to encompass the peak of corticosterone release by CT rats [37] and determine the inhibition of the HPA axis in GC group. The rats were anesthetized with thiopental (40 mg/kg; i.p.), weighted, and had their nasal–anal length measured (Day 28). The euthanasia was performed by decapitation for truncal blood collection, and serum was stored at −80 °C for hormonal and biochemical analysis. Afterward, the following tissues were dissected, weighed, processed as described below, or stored at −80 °C for later analysis: adrenal glands (right and left); mesenteric (meWAT), perirenal (prWAT), retroperitoneal (rpWAT), epididymal (epWAT), subcutaneous inguinal (scWAT), and interscapular brown (BAT) ATs; gastrocnemius (G), soleus (S), and extensor digitorum longus (E) muscles, tibia bone, hypothalamus, and the liver. The experiment was not blinded, the researches involved in the sample collection knew the identification of each rat. Each step was done by the same person to avoid possible differences in the dissection delimitation or weighting criteria.

The data about food intake, adrenal glands, ATs, muscles, and liver mass were expressed in grams (g) or milligrams (mg) of tissue, or relative to 100 g of body weight (g or mg/100 g b.w.). The change in body weight was calculated by the difference between the initial and final body weight in grams. The WAT mass was calculated as the sum of the five WAT depots analyzed in this study and presented as WAT or WAT + BAT, relative to 100 g of body weight (g/100 g b.w.). The feed efficiency was calculated by the ratio between weight change (g) and food intake (g). Additionally, 6 and 9 samples from CT and GC groups, respectively, were randomly selected previously to euthanasia to assess the G, S, and E dry muscle weight, and the remaining muscle samples were frozen for further analysis not presented in this study. For dry weight measurement, muscles were placed at 37 °C for 3 days, and then weighted.

### 2.5. Hormonal and Biochemical Analysis

The blood serum collected at euthanasia was used for the measurements of corticosterone (#501320, Cayman Chemical, Ann Arbor, MI, USA, glucose (#133, Labtest, Lagoa Santa, MG, Brazil), lactate (#138, Labtest, Lagoa Santa, MG, Brazil), insulin (#A05105, Bertin Bioreagent, Montigny-le-Bretonneux, France), leptin and adiponectin (#RADPCMAG-82K-07, Millipore, Darmstadt, Germany), non-esterified fatty acids-NEFA (NEFA-HR (2), Wako, Neuss, Germany), triglycerides (#87, Labtest, Lagoa Santa, MG, Brazil), total cholesterol (#76, Labtest, Lagoa Santa, MG, Brazil), HDL-cholesterol (#13, Labtest, Lagoa Santa, MG, Brazil), alanine aminotransferase-ALT (#1008, Labtest, Lagoa Santa, MG, Brazil), and aspartate aminotransferase-AST (#109, Labtest, Lagoa Santa, MG, Brazil). The serum concentration of LDL cholesterol was estimated by the Friedewald equation [38], as well as VLDL cholesterol. The HOMA-IR index was calculated as previously described [39] but using the average of fasting blood glucose and insulin product from CT rats as a correction factor, instead of the 22.5 used for humans.

For liver triglycerides assessment, lipids were extracted from liver samples with chloroform-methanol by the Folch method [40], and the triglycerides in the lipid extract were determined by enzymatic assay (#87, Labtest, Lagoa Santa, MG, Brazil) and expressed by milligrams of triglycerides by 100 milligrams of the liver.

### 2.6. Histological Analysis

One of the adrenal glands (sagittal section), a section of the liver left medial lobe, and sections of the ATs were fixed in a solution of 10% formaldehyde in PBS (phosphate buffered saline; pH 7.4) for 24 h and submitted to histological processing as previously described [41]. The sections were stained with hematoxylin and eosin (HE) and captured at 100×, 200× or 400× magnification (DS-Ri2 microscope, Nikon, Tokyo, Japan), as indicated in the Figures captions. 

The adipocyte volume for each WAT deposit in isolated adipose cells was also evaluated. For this purpose, adipocytes of meWAT, prWAT, rpWAT, epWAT, and scWAT were isolated [42], fixed in a solution of 4% formaldehyde in PBS. For the adipocytes image capture, the cells suspension was added to a glass slide, and the pictures were clicked at 100× magnification. The adipocyte volume in picoliters(pL) was calculated as previously described [43] and was presented as frequency distribution for each group and WAT territory. All morphological analyses were performed using Motic Image Plus 3.0 software (Motic^®^ Instruments, Schertz, TX, USA).

BAT slides were also immunostained with UCP1 antibody, performed in Leica Bond Max IHC (Leica Biosystems, Nussloch, Germany). The slides were submitted to deparaffinization and antigen recovery by heat (#AR9640, Leica Biosystems, Nussloch, Germany) for 20 min. Slides were then placed in a protein block (#x0909, DAKO, Carpinteria, CA, USA) for 10 min, followed by anti-UCP1 (#ab10983, 1:1000, Abcam, Waltham, MA, USA) incubation for 60 min. The detection was performed using Bond Refine Polymer (#DS9800, Leica Biosystems, Nussloch, Germany). The slides were dehydrated, cleaned, and covered. Images were captured using the Ks 300 Imaging System 3.0 (Carl Zeiss Vision GmbH, Aalen, Germany) at 100× magnification.

### 2.7. RNA Isolation and Gene Expression Analysis

For the gene expression, 6 samples (hypothalamus) and 7–12 samples (adipose tissue) for each group were randomly selected. The remaining samples were frozen for further analysis not presented in this study. The final number of samples in gene expression for CT and GT groups were, respectively: 12-12 (meWAT), 12-11 (prWAT), 8-11 (rpWAT), 11-11 (epWAT), 7-11 (scWAT), and 8-8 (BAT).

Hypothalamus and ATs RNA were extracted following the TRIzol Reagent^®^ (#15596026, Invitrogen, Waltham, MA, USA) and purified by PureLinkTM RNA Mini kit (#121830-18A, Ambion by Life Technologies, Carlsbad, CA, USA). Assessment of RNA quantity and quality was performed with Epoch Microplate Spectrophotometer (Biotek, Winooski, VT, USA). All samples were treated with RNase-free DNase I (#18068, Invitrogen, Waltham, MA, USA) before cDNA synthesis. For reverse transcription, 2 μg of hypothalamus RNA was used with SuperScript^®^ II Reverse Transcriptase (#18064, Invitrogen, Waltham, MA, USA) and random primers p(dN)6 (Sigma-Aldrich, San Louis, MO, USA), while in ATs, RNA was used with SuperScript^®^ III Reverse Transcriptase (#18080, Invitrogen, Waltham, MA, USA) and random primer (#48190, Invitrogen, Waltham, MA, USA). 

The qPCR of hypothalamus samples was performed using the 7500 Fast Real-Time PCR System (Applied Biosystems, Waltham, MA, USA) with SYBR Green PCR Master Mix (Applied Biosystems, Waltham, MA, USA). The primers used are described in Table 1.

The qPCR reactions of ATs samples were performed using TaqMan^®^ Gene Expression (Applied Biosystems, Waltham, MA, USA) for each interest gene: *Abhd5* (Rn01446981_m1); *Acaca* (Rn00573474_m1); *Acly* (Rn00566411_m1); *Actb* (Rn00667869_m1); *Adrb1* (Rn00824536_s1); *Adrb2* (Rn00560650_s1); *Adrb3* (Rn00565393_m1); *Agpat1* (Rn01525981_g1); *Agpat2* (Rn01438505_m1); *Akt1* (Rn00583646_m1); *Aqp7* (Rn00569727_m1); *B2m* (Rn00560865_m1); *Cd36* (Rn01442639_m1); *Cidea* (Rn04181355_m1); *Dgat1* (Rn00584870_m1); *Dgat2* (Rn01506787_m1); *Dio2* (Rn00581867_m1); Fabp4 (Rn00670361_m1); *Fasn* (Rn00569117_m1); *G0s2* (Rn01412529_g1); *G6pd* (Rn01529640_g1); *Gk* (Rn00577740_m1); *Gpam* (Rn00568620_m1); *Gpd1* (Rn00573596_m1); *Hsd11b1* (Rn00567167_m1); *Insr* (Rn00690703_m1); *Irs1* (Rn02132493_s1); *Irs2* (Rn01482270_s1); *Ldhb* (Rn00754925_m1); *Lipe* (Rn00689222_m1); *Lpl* (Rn00561482_m1); *Me1* (Rn00561502_m1); *Mgll* (Rn00593297_m1); *P2rx5* (Rn00589966_m1); *Pck1* (Rn01529014_m1); *Pde3b* (Rn00568191_m1); *Pik3cg* (Rn01769524_m1); Pik3r1* (Rn01644964_m1); *Plin1* (Rn00558672_m1); *Pnpla2* (Rn01479969_m1); *Pparg* (Rn00440945_m1); *Ppargc1a* (Rn00580241_m1); *Prdm16* (Rn01516224_m1); *Prkaca* (Rn01432300_g1); *Prkacb* (Rn01748540_g1); *Rpl37a* (Rn02114291_s1); *Scd* (Rn06152614_s1); *Scd2* (Rn00821391_g1); *Slc16a1* (Rn00562332_m1); *Slc16a7* (Rn00568872_m1); *Slc2a1* (Rn01417099_m1); *Slc2a4* (Rn00562597_m1); *Slc3a2/Pat2* (Rn00595142_m1); *Tfrc* (Rn01474695_m1); *Tmem26* (Rn01428021_m1); *Ucp1* (Rn00562126_m1); *Zic1* (Rn00575376_m1). The reactions were performed in the StepOnePlusTM Real-Time PCR System (Applied Biosystems, Waltham, MA, USA), using TaqMan^®^ Universal PCR Master Mix (#4304437, Applied Biosystems, Waltham, MA, USA). 

The gene expression analysis was performed by relative quantification (2^−∆∆CT^), and the housekeeping gene was used according to tests performed on each tissue. For the hypothalamus, we used the geometric mean of *Actb*, *Gapdh*, and *Ppia.* For the ATs samples, five different genes were tested as housekeeping-*Actb*, *B2m*, *Hprt1*, *Rpl37a*, and *Tfrc*; and the gene without variation between the CT and GC groups was choose to the normalizations, as follow: *Actb* on BAT, prWAT, and rpWAT; *B2m* on epWAT; *Rpl37a* on meWAT; and *Tfrc* on scWAT samples. 

### 2.8. BAT Oxidative and Lipogenic Capacity

The citrate synthase maximal activity in BAT samples was evaluated as previously described [44]. Samples were diluted (1:100) and the linear variation of absorbance in the function of time was used to calculate the maximal activity of the enzyme, and the results were normalized by µg of protein. Basal oxidative capacity to D-[U-^14^C]-glucose (#CFB96, Amersham Biosciences, Buckinghamshire, UK) and [1-^14^C]-palmitic acid (#CFA23, Amersham Biosciences, Buckinghamshire, UK) in BAT samples (50 mg) was performed according to Sertié et al. [45], modified to tissue, and expressed as ^14^CO_2_ nmol/100 mg of BAT. The D-[U-^14^C]-glucose incorporated into lipids was performed to assess the lipogenic capacity as previously described [46], modified to BAT samples, and the results are expressed as nmol/100 mg of BAT. 

### 2.9. In Vivo Body Temperature Analysis

Corporal (dorsal and ventral) thermal images were captured from anesthetized CT and GC rats on days 0 and 28th, between 12 < ZT < 13, using the FLIR^®^ E53 camera (Teledyne FLIR, Wilsonville, OR, USA). The images were analyzed by the FLIR^®^ Thermal Studio software version 1.9.23 (Teledyne FLIR, Wilsonville, OR, USA), and the results were expressed as the mean temperature (°C) of the corporal and BAT area (interscapular region).

### 2.10. Statistical Analysis

All data were tested to distribution (Shapiro–Wilk test), and homogeneity of variances (F test). For comparisons between CT and GC groups, unpaired Student *t*-test or Mann–Whitney test were used, as indicated in the figure captions. When the F test was significant, Welch correction was performed after the Student *t*-test. For nasal–anal length comparison on days 0 and 28th, a Two-Way ANOVA was used, with Bonferroni posthoc due to significant interaction. For correlation analyses between plasma leptin levels and adipocytes volume, after the data showed normal distribution, Pearson’s test was used. When there was a correlation, linear regression was used to draw the line that represents the correlation. The level of significance assumed was 5% (*p* < 0.05).

## 3. Results

### 3.1. Inhibition of the HPA Axis

**Figure 1:**
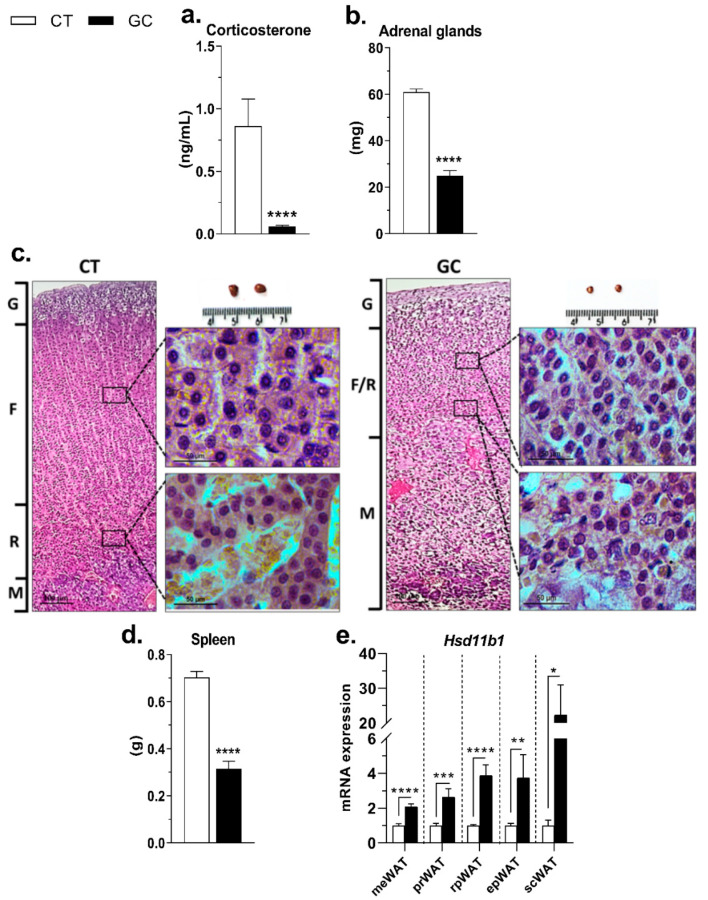
**Glucocorticoid continuous administration promoted inhibition of the HPA axis.** (**a**) Serum corticosterone, (**b**) adrenal gland mass, and (**c**) histological sections; (**d**) spleen mass, and (**e**) *Hsd11b1* gene expression in mesenteric—meWAT, perirenal—prWAT, retroperitoneal—rpWAT, epididymal—epWAT, and subcutaneous inguinal—scWAT of control (CT), and glucocorticoid-treated rats (GC). Data are mean ± SEM of 13 (CT) and 17 (GC) rats (**a**,**b**,**d**), and 7–12 (CT) 11-12 (GC) samples (**e**). * *p* < 0.05; ** *p* < 0.01; *** *p* < 0.001 and **** *p* < 0.0001 vs. CT (Unpaired Student *t*-test; Mann–Whitney test in **a** and **d**). (**c**) HE staining—100× and 400× magnification. G: Glomerulosa zone; F: Fasciculata zone; R: Reticularis zone, and M: Medulla.

Chronic GC administration for 4 weeks promoted HPA axis inhibition, as indicated by a 94% reduction in serum corticosterone (Figure 1a), and a 59% reduction of adrenal gland mass (Figure 1b) in GC rats. The reduction of adrenal mass, mainly due to fasciculate (F) and reticular (R) zone atrophy, was confirmed by the histological analysis (Figure 1c). The treatment also promoted a reduction in spleen mass (Figure 1d). The expression of *Hsd11b1* was increased in all WATs, probably due to the absence of corticosterone inhibition/regulation (Figure 1e).

### 3.2. Hypercortisolism and Metabolic Changes

**Figure 2:**
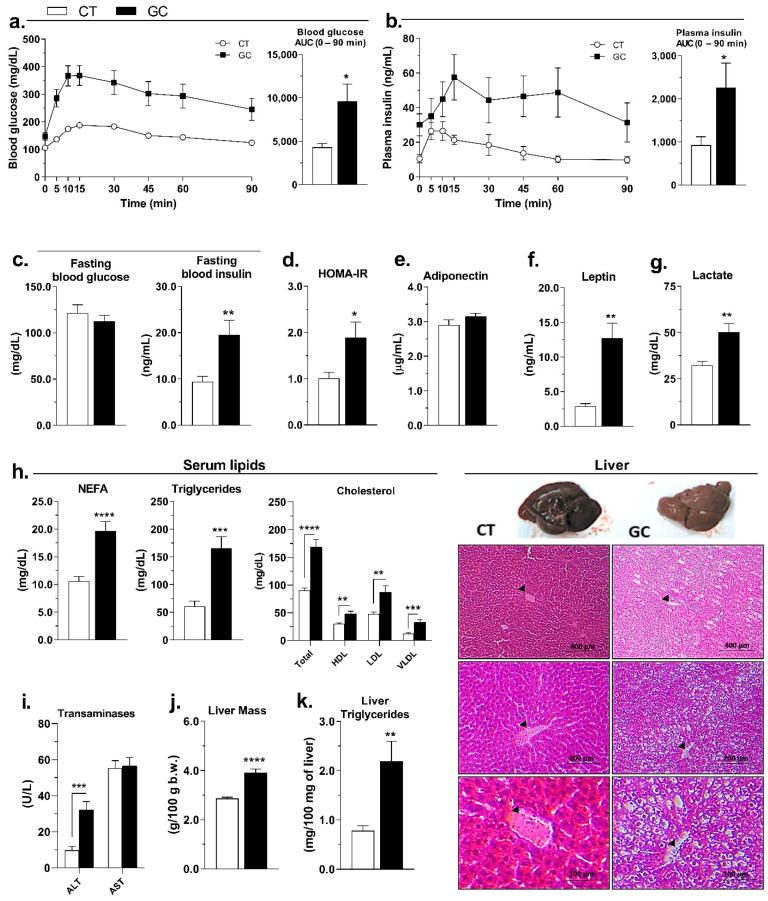
Chronic iatrogenic hypercortisolism promotes glucose intolerance, insulin resistance, dyslipidemia, and increase of hepatic lipid content. (**a**) Blood glucose and (**b**) plasma insulin during oGTT test; (**c**) 12h fasted blood glucose and insulin; (**d**) HOMA-IR; serum (**e**) adiponectin, (**f**) leptin; (**g**) lipids-NEFA, triglycerides, total cholesterol, HDL, LDL, and VLDL-cholesterol fractions; (**h**) lactate, and the (**i**) transaminases ALT and AST; (**j**) liver mass, (**k**) triglycerides content, and histological sections of control (CT), and glucocorticoid-treated (GC) rats. HE staining; 100×, 200×, and 400× magnification; the black arrows indicates the central vein. Data are mean ± SEM of 13 (CT) and 17 (GC) rats/samples. * *p* < 0.05; ** *p* < 0.01; *** *p* < 0.001, and **** *p* < 0.0001 vs. CT (unpaired Student *t*-test, Mann–Whitney test in **b**-AUC, and **c**-fasting glucose).

The chronic GC exposure promoted glucose intolerance, as evidenced by the incremental area under the curve (AUC) comparison (Figure 2a). We also evaluated the insulinemia during oGTT and, as shown in Figure 2b, the GC-treated rats appeared to have an initial delay in insulin secretion (5 to 15 min), and a hyperinsulinemic profile was observed during the test, as confirmed by AUC analysis (Figure 2b). No difference was observed in 12 hours-fasting glycemia, but GC rats showed hyperinsulinemia in this condition (Figure 2c), resulting in increased HOMA-IR (Figure 2d).

The GC excess did not change serum adiponectin levels (Figure 2e) but promoted the increase in leptin (Figure 2f), NEFA, triglycerides, cholesterol (Figure 2g), and lactate (Figure 2h) compared to the CT group. Additionally, the treatment increased serum levels of ALT but did not change AST (Figure 2i). GC-treated rats also showed an increase in liver mass, with changes in hepatocytes morphology (Figure 2j) and increase in liver triglycerides content (Figure 2k).

**Figure 3:**
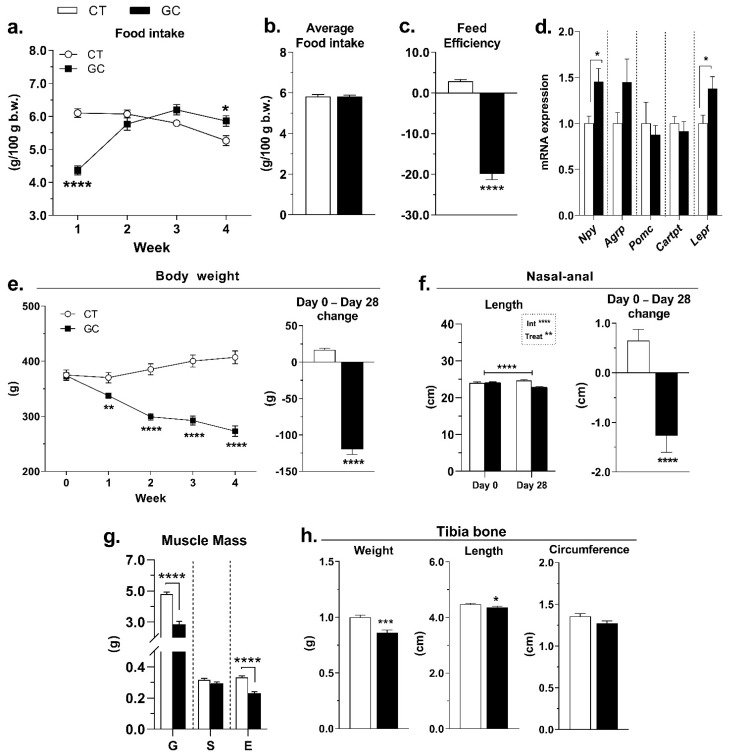
**Food intake and body changes in response to GC excess**. (**a**) Daily food intake per week; (**b**) average 28 days daily food intake; (**c**) feed efficiency; (**d**) hypothalamic gene expression of *Npy*, *Agrp*, *Pomc*, *Cartpt*, and *Lepr*; (**e**) body weight change; (**f**) nasal–anal length; (**g**) gastrocnemius—G, soleus—S, and extensor digitorum longus—E muscle mass; and (**h**) tibia bone weight, length, and circumference of control (CT), and glucocorticoid-treated (GC) rats. Data are mean ± SEM of 13 (CT) and 17 (GC) rats/samples (**a**–**c**,**e**–**h**), and 6 (CT) and 6 (GC) samples (**d**). * *p* < 0.05; ** *p* < 0.01; *** *p* < 0.001 and **** *p* < 0.0001 vs. CT (unpaired Student *t*-test; Mann–Whitney test in **h**-length). (**f**) **** *p* < 0.0001 (Repeated Measures Two-Way ANOVA with Bonferroni’s post hoc test).

The food intake changed along with the treatment in GC rats. While food intake was reduced in the first week, in the last week GC rats exhibited increased food intake compared to the CT group (Figure 3a). When the entire period is analyzed, the average food intake did not change in response to GC treatment (Figure 3b), but feed efficiency was negative (Figure 3c). We also evaluated the hypothalamic gene expression of *Npy, Agrp, Pomc, Cartpt*, and Lepr, in which only *Npy* and *Lepr* were increased by GC administration (Figure 3d). Other genes were analyzed in the hypothalamus, but they did not differ between CT and GC groups (Figure S1a).

GC rats showed accentuated body weight loss (Figure 3e and Figure S1c). To better understand it, we also evaluated the nasal–anal length as three different types of skeletal muscle and the tibia bone, and GC rats showed a reduction of CNA at the end of the treatment (Figure 3f), as well as the gastrocnemius and EDL muscle mass, while soleus muscle showed no difference in mass (Figure 3g and Figure S1b). The treatment also promoted a decrease in the tibia bone weight and length but did not change tibia circumference (Figure 3h).

### 3.3. GC-Induced Adipose Tissue Redistribution

**Figure 4:**
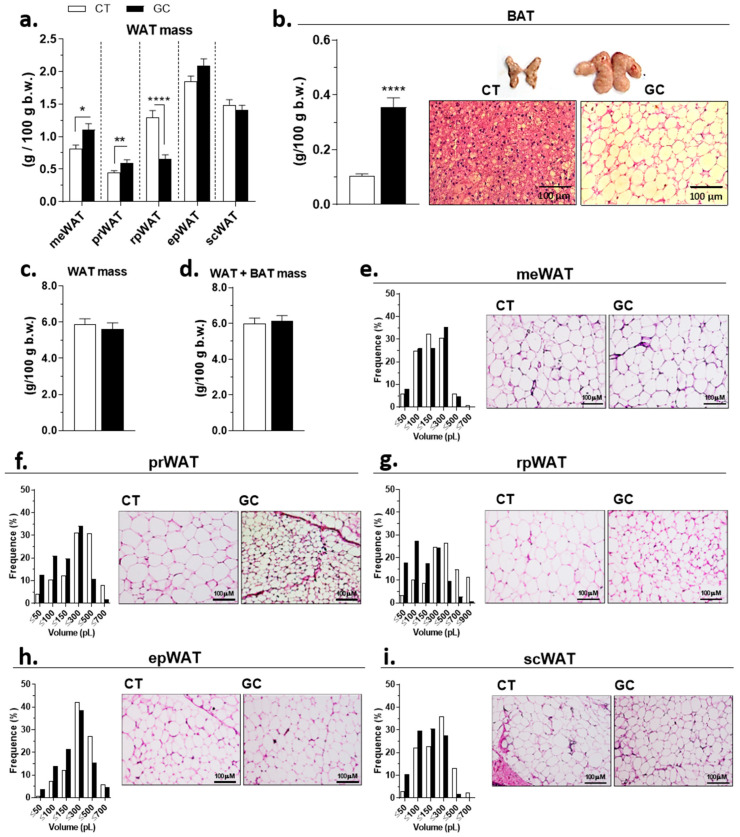
**Adipose tissue redistribution after chronic glucocorticoid treatment.** (**a**) adipose mass of mesenteric—meWAT, perirenal—prWAT, retroperitoneal—rpWAT, epididymal—epWAT, and subcutaneous inguinal—scWAT; (**b**) interscapular brown adipose tissue—BAT mass and histological sections; (**c**) WAT mass; (**d**) WAT + BAT mass; frequency distribution of adipocyte volume (pL) and histological sections of (**e**) meWAT, (**f**) prWAT, (**g**) rpWAT, (**h**) epWAT, and (**i**) scWAT of control (CT), and glucocorticoid-treated (GC) rats. HE staining; 100× magnification. Data are mean ± SEM of 13 (CT) and 17 (GC) rats/samples. * *p* < 0.05; ** *p* < 0.01, and **** *p* < 0.0001 vs. CT (unpaired Student *t*-test).

GC-treated rats showed AT redistribution: an increase in two visceral depots—meWAT and prWAT, and expressive reduction of rpWAT—but no significant difference was observed in epWAT and scWAT (Figure 4a). The interscapular BAT of GC rats’ mass was 3.4 fold bigger than the CT group, and an unilocular phenotype was also observed (Figure 4b). GC treatment did not change the WAT mass sum of all five different depots analyzed in this study (Figure 4c), as no difference was observed when considering WAT + BAT mass (Figure 4d). 

Despite the increase in meWAT mass, there were no changes in the adipocytes volume frequency (Figure 4e). An increase in the frequency of adipocytes with smaller volumes was observed in prWAT (Figure 4f), rpWAT (Figure 4g), epWAT (Figure 4h), and scWAT (Figure 4i), even though the difference in mass was among the fat pads. In the CT group, there was a correlation between the serum leptin and adipocyte volume in all WAT deposits, but this effect was abolished by GC treatment (Figure S2).

### 3.4. Direct and Permissive Actions by GCs in Gene Expression of WAT Lipolysis and Lipogenesis Pathways

**Figure 5:**
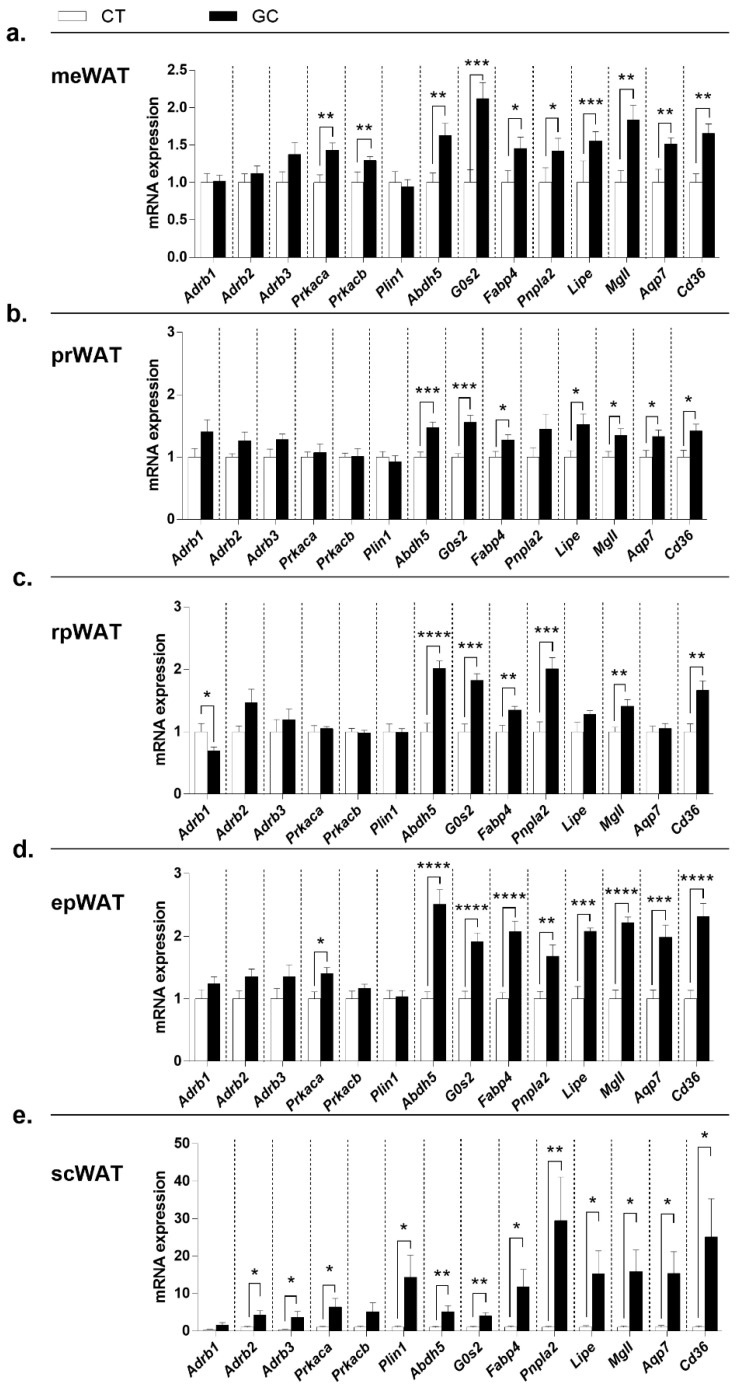
**Gene expression of the lipolytic pathway in white adipose tissue.** (**a**) mesenteric—meWAT; (**b**) perirenal—prWAT; (**c**) retroperitoneal—rpWAT; (**d**) epididymal—epWAT; and (**e**) subcutaneous inguinal—scWAT. Genes of beta-adrenergic receptors—*Adrb1, Adrb2*, and *Adbr3;* catalytic subunits of PKA—*Prkaca* and *Prkacb*; perilipin in white mature adipocytes—*Plin1*; lipase cofactors—*Abdh5, G0s2*, and *Fabp4*; lipases—*Pnpla2, Lipe*, and *Mgll*; lipolytic products channel and transporter—*Aqp7* and *Cd36*—of control (CT; n = 7–12), and glucocorticoid-treated (GC; n = 11–12) rats. Data are mean ± SEM. * *p* < 0.05; ** *p* < 0.01, *** *p* < 0.001, and **** *p* < 0.0001 vs. CT (unpaired Student *t*-test; Mann–Whitney test in (**a**) (*Adrb2, Adrb3, Fabp4, Abdh5, Mgll*, and *Aqp7*), (**b**) (*Adrb3* and *Fabp4*), (**c**) (*Adrb2* and *Adrb3*), (**d**) (*Adrb2, Adrb3*, and *Prkacb*), and **e** (all, except *G0s2*)).

The beta-adrenergic receptors’ gene expression diverged among the analyzed WAT (Figure 5). The meWAT (Figure 5a), prWAT (Figure 5b), and epWAT (Figure 5d) did not show any difference between the groups, while in rpWAT (Figure 5c), *Adrb1* decreased expression in GC rats, and *Adrb2* and *Adrb3* increased in scWAT (Figure 5e). *Prkaca* increased in meWAT (Figure 5a), epWAT (Figure 5d), and scWAT (Figure 5e), whereas *Prkacb* only increased in meWAT (Figure 5a) of GC rats. *Plin1* is overexpressed in scWAT (Figure 5e) and the other lipolytic genes—*Abdh5, G0s2, Fabp4, Pnpla2, Lipe, Mgll, Aqp7,* and *Cd36*—showed higher mRNA expression in GC rats, except for *Pnpla2* in prWAT (Figure 5b), and *Lipe* and *Aqp7* in rpWAT (Figure 5c). 

**Figure 6:**
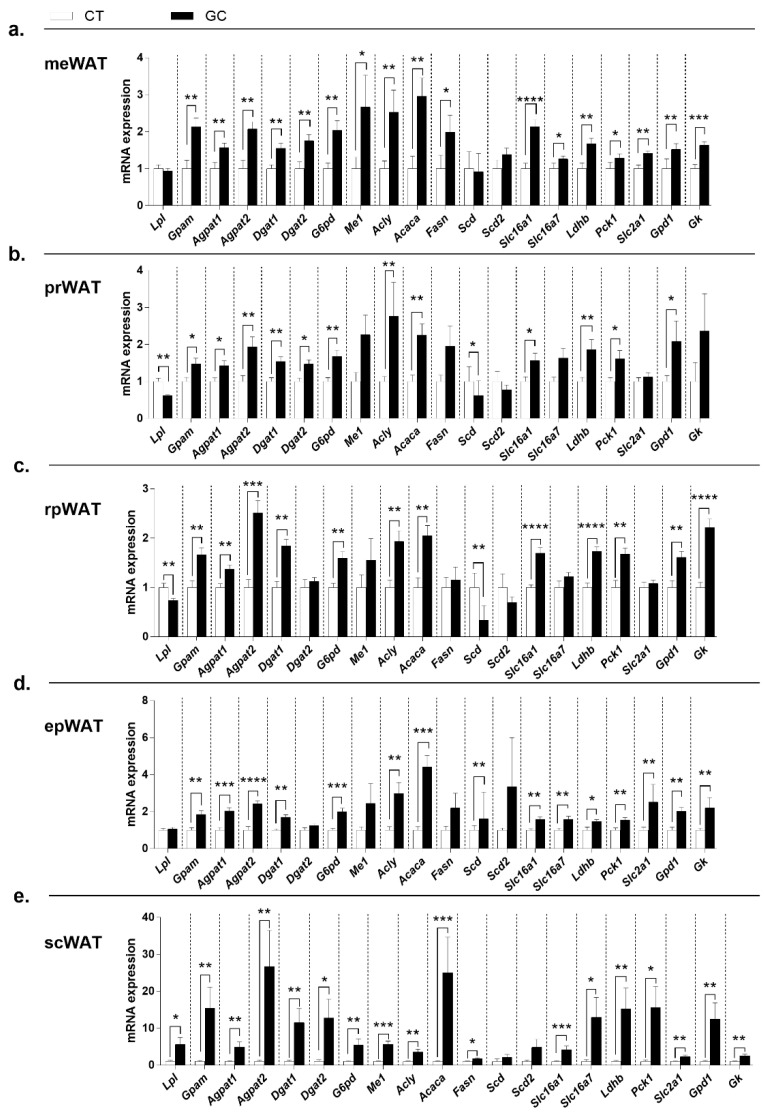
**Gene expression of the lipogenic pathway in white adipose tissue.** (**a**) mesenteric—meWAT; (**b**) perirenal—prWAT; (**c**) retroperitoneal—rpWAT; (**d**) epididymal—epWAT; and (**e**) subcutaneous inguinal. Genes of lipoprotein lipase—*Lpl*; enzymes of fatty acids esterification—*Gpam, Agpat1, Agpat2, Dgat1,* and *Dgat2*; cytosolic NADPH recyclers—*G6pd* and *Me1*; enzymes of lipogenesis de novo—*Acly, Acaca*, and *Fasn;* fatty acid desaturases—*Scd* and *Scd2*; monocarboxylate transporters—*Slc16a1* and *Slc16a7*; lactate dehydrogenase—*Ldhb*; the key enzyme of glyceroneogenesis—*Pck1*; glucose transporter 1—*Slc2a1*; the last enzyme of glycerol-3-phosphate generation glycolytic pathway and glyceroneogenesis—*Gpd1*; and the glycerol kinase—*Gk*—of control (CT; n = 7–12), and glucocorticoid-treated (GC; n = 11–12) rats. Data are mean ± SEM. * *p* < 0.05; ** *p* < 0.01; *** *p* < 0.001, and **** *p* < 0.0001 vs. CT (unpaired Student *t*-test; Mann–Whitney test in (**a**) (*Gpam, G6pd, Me1, Acly, Acaca, Fasn, Scd, Scd2* and *Gpd1*), (**b**) (*Agpat1, Dgat2, G6pd, Me1, Acly, Acaca, Scd, Slc16a7,* and *Gk)*, (**c**) (*Me1, Fasn, Scd,* and *Slc2a1*), (**d**) (*Agpat2, G6pd, Acly,* and *Ldhb*), and (**e**) (*Lpl, Agpat2, Dgat1, Dgat2, Acaca, Scd2, Slc16a1, Slc16a7, Pck1, Slc2a1,* and *Gpd1*)).

The same overexpressed profile was observed concerning lipogenic genes (Figure 6). All WATs increased the expression of *Gpam, Agpat1, Agpat2, Dgat1, G6pd, Acly, Acaca*, *Slc16a1, Ldhb, Pck1*, and *Gpd1*, but some genes showed different patterns in the WATs.

In the meWAT, there was no change in *Lpl, Scd,* and *Scd2,* but the expression of *Dgat2*, *Me1, Fasn, Slc16a7, Slc2a1*, and *Gk* were increased in GC group (Figure 6a). Additionally, the treatment promoted the reduction of *Lpl,* and *Scd* expression in prWAT and rpWAT, but did not change the expression of *Me1, Fasn, Scd2, Slc16a7*, and *Slc2a1* in these deposits (Figure 6b,c). Therefore, in the prWAT the GC excess did not change *Gk*, but increased *Dgat2* expression (Figure 6b), while in rpWAT there was no change in *Dgat2*, but increased *Gk* expression (Figure 6c). No differences were observed in the expression of *Lpl, Dgat2, Me1, Fasn*, and *Scd2* in the epWAT (Figure 6d), also as *Scd* and *Scd2*, in the scWAT (Figure 6e), but the expression of *Slc16a7, Slc2a1*, and *Gk* increased in both deposits in the GC group. In addition, *Scd* expression was higher in epWAT, and *Lpl, Dgat2, Me1*, and *Fasn* expression were increased in scWAT (Figure 6d,e).

Regarding the insulin pathway, the only gene that decreased expression was *Pik3cg* in prWAT (Figure S3b) and rpWAT (Figure S3c). *Insr, Irs1, Irs2, Pik3r1, Akt1, Pde3b*, and *Slc2a4* showed elevated mRNA expression in GCs rats or did not change (Figure S3). *Gpr81* expression was also increased in meWAT, epWAT, and scWAT, but no difference was observed in prWAT and rpWAT (Figure S3g).

### 3.5. BAT Whitening in Response to GC Excess

**Figure 7:**
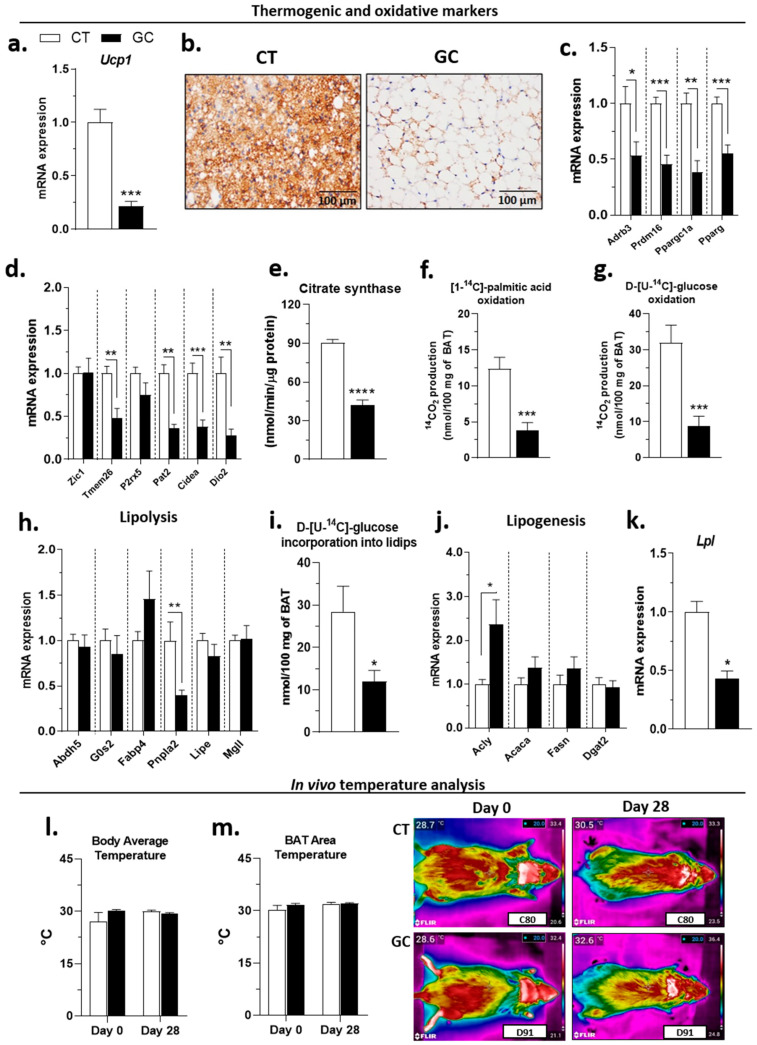
**BAT gene and functional changes after chronic GC exposure.** (**a**) *Ucp1* gene expression; (**b**) UCP1 immunohistochemistry; (**c**) *Ucp1* expression regulators—*Adrb3, Prdm16, Ppargc1a*, and *Pparg*; (**d**) brown adipocytes markers—*Zic1, Tmem26, Pat2, P2rx5, Cidea*, and *Dio2*; (**e**) citrate synthase maximal activity; oxidation of (**f**) [1-^14^C]-palmitic acid and (**g**) D-[U-^14^C]- glucose; (**h**) lipolysis pathway genes—*Abdh5, G0s2, Fabp4, Pnpla2, Lipe,* and Mgll; (**i**) incorporation of D-[U-C^14^]- glucose into lipids; (**j**) lipogenic pathway genes—*Acly, Acaca, Fasn*, and *Dgat2*; (**k**) *Lpl* expression; *in vivo* temperature analysis of (**l**) body and (**m**) BAT area on days 0 and 28^th^ of control (CT), and glucocorticoid-treated (GC) rats. (**b**) UCP1 staining: 100× magnification. Data are mean ± SEM of 8 (CT) and 8 (GC) samples (**a**,**c**,**d**,**h**,**j**,**k**), 8 (CT) and 9 (GC) samples (**e**,**f**,**g**,**i**), and 10 (CT) and 11 (GC) rats (**l**,**m**). * *p* < 0.05, ** *p* < 0.01, *** *p* < 0.001, and **** *p* < 0.0001 vs. CT (unpaired Student *t*-test; Mann–Whitney test in (**c**)-*Adrb3* and *Ppargc1a*).

In addition to the increase in mass and unilocular phenotype (Figure 3b), the BAT of GC rats showed a decrease in the *Ucp1* gene (Figure 7a) and protein, as observed in the UCP1 immunostaining (Figure 7b). The gene expression of the pathway *Adrb3-Prdm16-Ppargc1a-Pparg*, which regulates *Ucp1* expression, was also reduced in GC rats BAT (Figure 7c), as well as the expression of the BAT markers *Zic1, Tmem26, Pat2, Cidea*, and *Dio2* (Figure 7d). The activity of citrate synthase (Figure 7e) and the oxidative capacity for palmitic acid (Figure 7f) and glucose (Figure 7g) were also reduced in the BAT of GC-treated rats.

In the lipolytic pathway, GC treatment decreased the *Pnpla2* expression but did not change *Abdh5, G0s2, Lipe, Fabp4*, and *Mgll* in the BAT (Figure 7h). The lipogenic capacity was measured by the incorporation of D-[U-^14^C]-glucose into the lipids, which was also reduced in the BAT of the GC group (Figure 7i), even the increase in *Acly* (Figure 7j). Therefore, no significant changes were observed in the other lipogenic genes—*Acaca, Fasn,* and *Dgat2* in the GC group (Figure 7j)—but the treatment reduced the *Lpl* expression (Figure 7k). GC administration did not change the insulin pathway genes evaluated—*Insr, Pik3r1, Akt1, Slc2a4,* and *Slc2a1*, except *Pik3cg*, which was reduced in GC-rats BAT (Figure S3f). The *Gpr81* expression was reduced in BAT of GC-rats (Figure S3g). 

Despite these changes in BAT, known for its thermogenic effects, there was no difference between CT and GC groups in body average temperature (Figure 7l) and BAT area temperature (Figure 7m) on days 0 or 28 of treatment.

## 4. Discussion

Hypercortisolism models are common, but few are focused on the GC effects in several AT deposits. Additionally, some methods can be more stressful to the animals (daily administration or manipulation), which can lead to results interferences, or even not assure the exact daily dose, as in the case of treatments by drinking water. The GCs effects depend on the dose, time of use, and individual parameters, with pediatric/young patients being more susceptible to severe side effects [47]. For this reason, we build an experimental design less stressful for the animals, which supports continuous and invariable delivery of GC in a dose used in previous animal studies [29] and also pharmacologically in humans [48]. We chose young adult rats (12 weeks old) [35] to avoid more possible interferences in another hormonal axis (e.g., sexual hormones in the pubertal phase, which can also affect the AT distribution). 

The most important characteristic of hypercortisolism/Cushing’s syndrome is the disruption of HPA axis control and loss of the circadian rhythm of GC production and release [5]. The intense reduction of corticosterone levels and adrenal cortical mass found in GC-treated rats due to the atrophy of the fasciculate and reticular zones of the adrenal cortex (Figure 1b,c) confirms the HPA axis inhibition and adrenal insufficiency [49]. The immunosuppressive effect of treatment was confirmed by the GC rats’ spleen mass (Figure 1d) [50].

GC excess induces several metabolic alterations, mainly in glucose and lipid metabolism [51,52]. One factor that could lead to insulin resistance, as observed in the present model (Figure 2a–d), is the increase in circulating NEFA, as shown in Figure 2g. This effect is probably by the permissive role of GC to the actions of the lipolytic hormones as catecholamines, which contributes to an exacerbated mobilization of NEFA from adipose depots and allows their ectopic accumulation in non-specialized tissues as the liver (Figure 2k), favoring the local and systemic insulin resistance [33]. The increased serum ALT corroborates with the increased liver mass, the morphological alterations, and with the increase of triglycerides content in this tissue (Figure 2i–k), suggesting damage to this vital organ in GC rats. Moreover, GCs also have direct hepatic actions, promoting the increase in the expression of the de novo lipogenesis key enzymes such as acetyl-CoA carboxylase and fatty acid synthase [53], and increasing the synthesis and secretion of VLDLs [54]. Together, these effects contribute significantly to the establishment of dyslipidemia, another characteristic of human Cushing syndrome reproduced in our animal model (Figure 2g). 

These metabolic changes could also be a result of GC effects on food intake. Even with the increased serum leptin and hypothalamic *Lepr* expression (Figure 2f and Figure 3d), GC rats showed higher food intake in the last week of treatment (Figure 3a). Indeed, GC rats exhibited upregulation of *Npy* expression in the hypothalamus (Figure 3d). These findings corroborate with previous studies, showing that GCs can increase the hypothalamic expression of orexigenic neuropeptides such as NPY and AgRP, promoting increased food intake [55,56]. However, the average food intake did not differ between the groups when the entire period is analyzed (Figure 3b) and, due to the intense loss of body weight (Figure 3e), the GC rats had negative feed efficiency (Figure 3c). In this sense, we hypothesize that, even with the same average food intake during the 28 days, the nutrients may have a different metabolic fate in the GC-treated rats.

In humans, weight gain and an increase in central adiposity are the most visible features of Cushing syndrome [6,57,58]. However, a previous study with excess of synthetic GC induced weight loss in rats [29]. In concordance, our GC-treated rats were visibly “smaller and skinny”, and showed a decrease in nasal–anal length compared to CT rats (Figure 3f). GC-rats showed loss of muscle mass in two of the three muscles analyzed (Figure 3g and Figure S1b) and reduced tibia mass (g) and length (cm) compared to CT rats. It highlights the difference in the body composition of these animals, which are caused by GC catabolic effects [59,60]. For this reason, we consider normalizing the AT tissue weights by 100g of body weight, aiming to get more accurate comparisons between the groups. 

WAT is the most prevalent AT in humans and rodents, and depending on the local accumulation, there is a risk to develop diseases [61,62]. The increase of visceral adipose tissues (VAT) is more related to metabolic changes than subcutaneous adipose tissue (SAT), even SAT being the predominant adipose site in the body [61,63]. Humans facing Cushing syndrome develop a central fat accumulation pattern, with an increase in VAT and loss of peripheral SAT [60,64]. A previous study of our group did not observe significant AT redistribution typical of Cushing’s syndrome [25]. In our model, despite the accentuated overall weight loss (Figure 3e), there was a notable visceral/BAT fat redistribution without changes in the relative amount of WAT/WAT+BAT in treated rats compared to the CT group (Figure 4a–d). Additionally, GC rats display a divergent frequency of adipocyte size (Figure 4e–i), with smaller adipocytes compared to CT rats, which could lead to an improvement in the metabolic condition of GC rats [65,66], but it did not occur. 

Small adipocytes present increased secretion of adiponectin, an insulin-sensitizing hormone [67], an effect not observed in our model (Figure 2e). Another adipokine, leptin, has the opposite pattern—its secretion is related to hypertrophic adipocytes or increased adipose mass [68,69]. Our model showed increased leptin levels even with smaller adipocytes and no difference in total adipose tissue mass (Figure 2f, Figure 4c, and Figure S2). All these data confirmed an AT remodeling in our model and reinforce the evidence of AT’s role or its dysfunction in Cushing’s syndrome pathology. 

GCs are known as lipolytic hormones in AT [1,4,22,70,71], and the increased serum concentrations of NEFA in GC-treated rats suggest an increase of lipolysis. Since the majority of adverse effects provoked by long-term GC use are genomic [19], we analyzed gene transcription of factors that could interfere directly with the lipolysis pathway, such as lipases (*Pnpla2*, *Lipe*, and *Mgll*) and their cofactors (*Abdh5*, *G0s2*, and *Fabp4*), or indirectly, like beta catecholamines receptors (*Adrb1*, *Adrb2*, and *Adrb3*), catalytic subunits of PKA (*Prkaca* and *Prkacb*), perilipin A (*Plin1*), and transporter/channel for products (*Cd36* and *Aqp7*). None of the changes in the expression of these genes could explain the WAT redistribution observed, since the pattern of expression was similar among the different deposits (Figure 5).

Another phenomenon that could explain the AT redistribution is lipogenesis. This term includes many pathways, such as the esterification of fatty acids in glycerol-3-phosphate (G3P). Proteins involved are encoded by *Gpam*, *Agpat1*, *Agpat2*, *Dgat1*, and *Dgat2* gene in WAT [72], and formation de novo of fatty acids (*Acly*, *Acaca*, and *Fasn* are the genes of key-enzymes [73,74], *G6pd* and *Me1* are genes of enzymes that recycle NADPH [73]). Additionally, *Scd* and *Scd2* are genes that encode proteins not directly involved with the de novo lipogenesis pathway, signaling its occurrence [75], and G3P generation pathways, *Gk*, *Pepckc*, and *Gpd1,* encode key-enzymes, and the other genes, *Slc16a1*, *Slc16a7*, *Ldhb*, and *Slc2a1*, are involved with proteins that manage substrates for these pathways [26,73,76,77]. Again, there was a pattern of overexpression for most of lipogenic genes in the WAT of GC-treated rats (Figure 6), and the same pattern were observed in the genes of the insulin cascade (Figure S3a–e)—the major lipogenic hormone [78,79]. Despite increased HOMA-IR, at the genomic level there is no proof of insulin resistance in the WAT of GC-treated rats [80]. Regardless of metabolic reprogramming occasioned by GC treatment and all the depots of WAT respond to the inhibition of the HPA axis, increasing the expression of *Hsd11b1* (Figure 1e), their actions are promiscuous and wide, not justifying the AT redistribution that appears to be one of the main causes of metabolic alterations. 

In the present experimental model, the BAT of GC-treated rats completely loses its well-known characteristics: there was an increase in mass, changes in morphology to an unilocular phenotype, reduced expression of genes related to thermogenesis/brown adipocytes markers, and most importantly, the decreased oxidative capacity (Figure 7a–g), which characterizes a whitening process [81,82]. Our results corroborate with some studies that have shown that GC excess leads to changes in the morphology and/or thermogenic function [83,84,85,86,87]. However, it is still unclear whether BAT dysfunctions are a cause or a consequence of the AT redistribution/obesity that occurs in Cushing’s syndrome, but the BAT whitening may also explain some metabolic changes in this model.

Among the factors that can promote BAT dysfunction in obesity is the reduction of vascularization and, consequently, the β-adrenergic activation pathway that regulates the BAT activity, favoring *Ucp1* expression [88,89,90], and stimulates the lipolytic pathway to mobilize fatty acids for oxidation [91]. Along with the decreased *Ucp1*, the BAT of the GC rats showed reduced expression of *Adrb3* and *Atgl*, which encodes the first enzyme of the lipolytic pathway (Figure 7c,h). Fatty acids are the primary energy substrate for thermogenesis in brown adipocytes [91], and the intracellular triacylglycerol stock is replenished mainly by uptake of circulating fatty acids derived from lipolysis [92], and in second place, by the de novo lipogenesis from glucose uptake [93]. However, GC rats also present reduced *Lpl* expression (Figure 7k), which may indicate reduced fatty acids uptake. This result, added to the decreased oxidative capacity and possible inhibition of the lipolytic pathway, certainly plays a role in the dyslipidemia presented by the GC rats (Figure 2g).

The unilocular phenotype of brown adipocytes of GC-treated rats reveals a significant accumulation of triacylglycerol, which could be a combination of the factors of decreased fatty acids oxidation/thermogenesis/lipolysis plus an increased lipogenic capacity in this fat. Although the *Acly* expression was increased, the de novo lipogenesis, measured by the glucose incorporation into lipids, was reduced in the BAT of GC rats (Figure 7i,j). Since there was no difference in the expressions of *Slc2a1* and *Slc2a4* between CT and GC groups (Figure S3f), is not possible to suppose that the GC excess did not affect the BAT glucose uptake. The glucose may have different fates in BAT than oxidation to CO_2_, as shown by our results. For example, a previous study showed that brown adipocytes convert a large amount of glucose to lactate [94], which is mostly exported to the circulation but can also serve as a substrate for lipogenesis [76,95], or regulate pathways such as lipolysis through the activation of GPR81 [96]. Such pathways require further studies in our model since GC rats have higher serum lactate compared to CT rats (Figure 2h), but decreased *Gpr81* in BAT, and only rpWAT, which decreased in mass, showed no alteration in the *Gpr81* expression (Figure S3g).

Besides the significant changes in BAT, GC treatment did not promote significant changes in body and BAT area temperature by the thermal imaging method we use in this study (Figure 7l,m). Given these results, we hypothesize that the BAT dysfunction is being compensated by the browning of WAT territories in GC rats, which is actually under investigation by our research group.

In conclusion, this experimental model of Cushing’s syndrome in rats reproduced several characteristics of this syndrome observed in humans, such as HPA axis inhibition, AT redistribution with an increase in VAT (meWAT and prWAT), and metabolic changes such as glucose intolerance, insulin resistance, dyslipidemia, and hepatic lipid accumulation. The AT redistribution and remodeling in response to GC excess showed more importance than the increase of AT mass per se. This pattern observed in AT cannot be explained just by GC regulation of gene transcription, and other mechanisms should be explored.

## Figures and Tables

**Table 1:** Primers used on the hypothalamus qPCR.

Gene	Primer Sequences (5′-3′)	
	Forward	Reverse
*Actb*	AGCCTGGATGGCTACGTACA	CCTCTGAACCCTAAGGCCAA
*Agrp*	AGGACTCGTGCAGCCTTACAC	GCAGAGGTGCTAGATCCACAGAA
*Cartpt*	CCGCCTTGGCAGCTCCTT	CCGAGCCCTGGACATCTACT
*Crh*	CCGATAATCTCCATCAGTTTCCTG	TGGATCTCACCTTCCACCTTCTG
*Gapdh*	CCGTTCAGCTCTGGGATGAC	GGGCAGCCCAGAACATCAT
*Hcrp*	AGGGAGAGGCAATCCGGAGAG	GCGGCCTCAGACTCCT
*Npy*	CCCTCAGCCAGAATGCCCAA	CCGCCCGCCATGATGCTAGGTA
*Lepr*	CCAGAAGAAGAGGACCAAATATCAC	ACTTAATTTCCAAAAGCCTGAAACA
*Ppia*	TATCTGCACTGCCAAGACTGAGT	CTTCTTGCTGGTCTTGCCATTCC
*Pmch*	CTTCTACGTTCCTGATGGACTT	ATGCTGGCCTTTTCTTTGTTT
*Pomc*	GCAAGCCAGCAGGTTGCT	ATAGACGTGTGGAGCTGGTGC
*Tnf*	GGTTGTCTTTGAGATCCATGC	TCTCAAAACTCGAGTGACAAGC

## Data Availability

The datasets generated during and/or analyzed during the current study are available from the corresponding author on reasonable request.

## Supplementary Materials

The following supporting information can be downloaded at: https://www.mdpi.com/article/10.3390/biomedicines10092328/s1, Figure S1. Hypothalamic gene expression of *Pmch*, *Hcrp*, *Crh*, and *Tnf*; and representative body images of CT and GC rats; Figure S2. Correlation between plasma leptin levels and adipocytes volume of meWAT; prWAT; rpWAT; epWAT; scWAT, or WAT and WAT + BAT mass of CT and GC rats; Figure S3. Expression of classic insulin pathway genes in meWAT; prWAT; rpWAT; epWAT; scWAT, and BAT; and *Gpr81* in all fat deposits of CT and GC rats.
