# Supplementary figures and images for: Dexamethasone-Induced Adipose Tissue Redistribution and Metabolic Changes: Is Gene Expression the Main Factor? An Animal Model of Chronic Hypercortisolism
Source: Biomedicines. 2022 Sep 19;10(9):2328. doi: 10.3390/biomedicines10092328 (PMC9496558; doi:10.3390/biomedicines10092328)

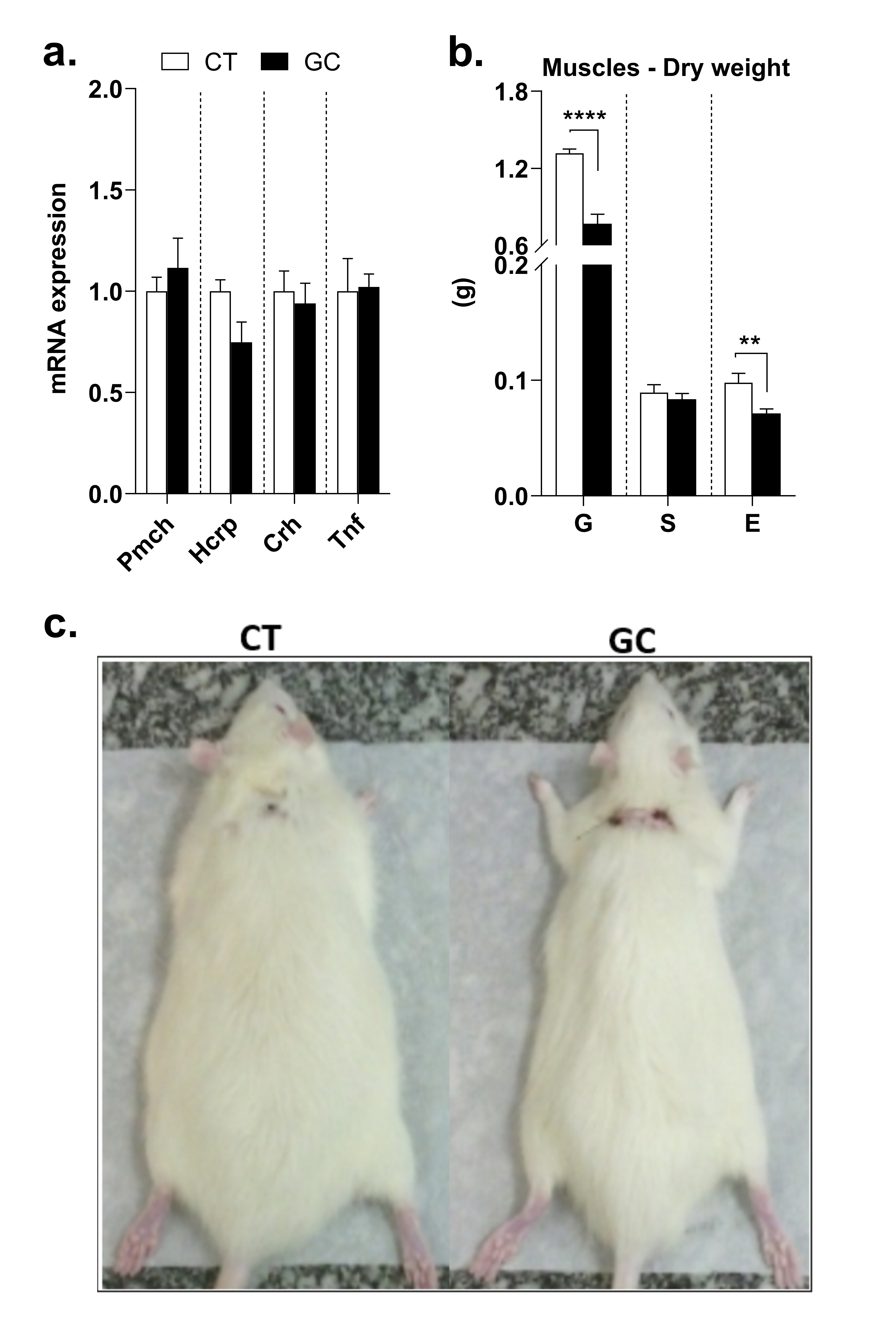

Supplement: Supplementary file 1 [file biomedicines-10-02328-s001.zip › Figure S1.tif]

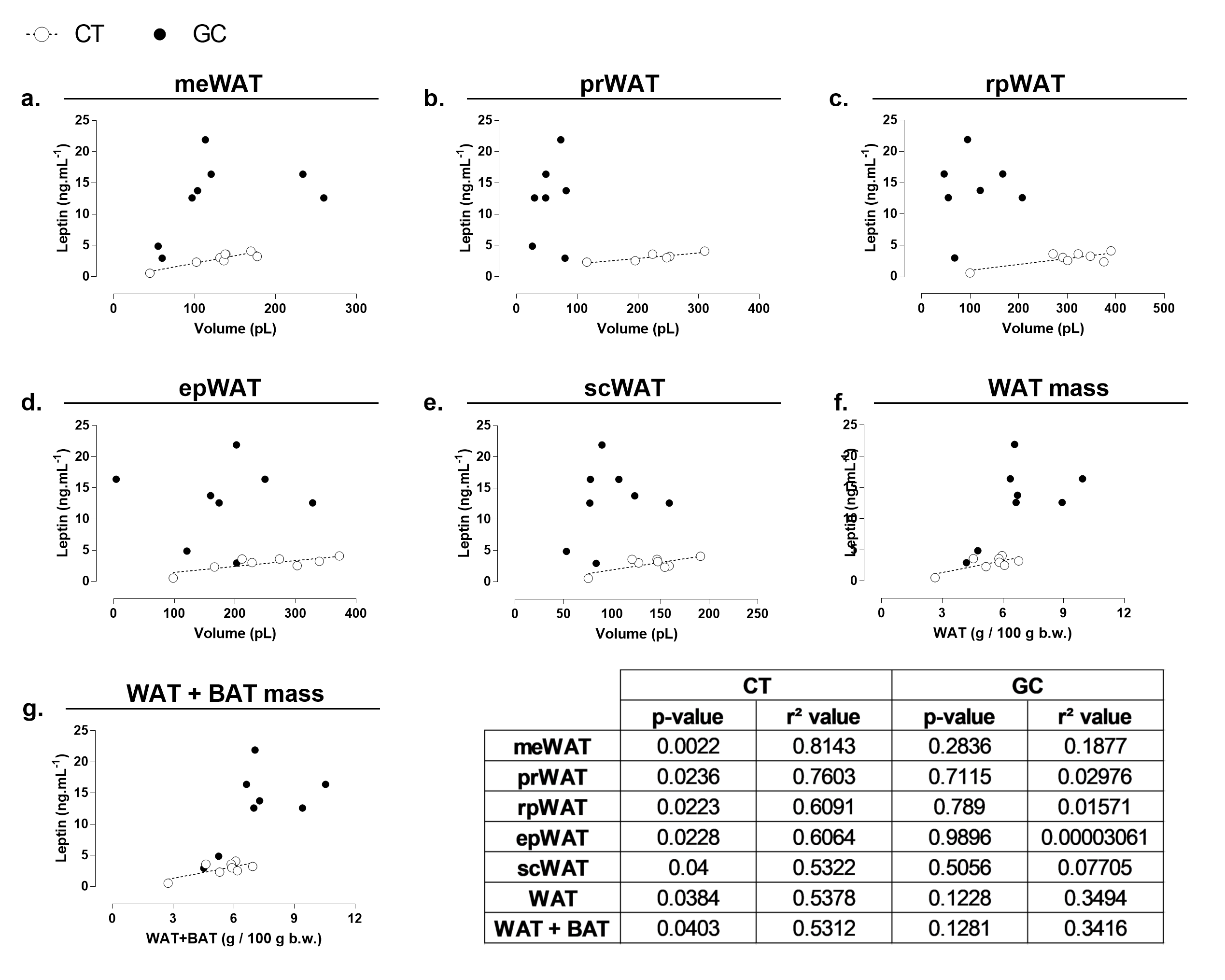

Supplement: Supplementary file 1 [file biomedicines-10-02328-s001.zip › Figure S2.tif]
